# Enhancing unsupervised bearing fault diagnosis through structured prediction in latent subspace

**DOI:** 10.1038/s41598-025-26013-0

**Published:** 2025-11-26

**Authors:** Chen Liu, Runshan Hu, Xuan Fang, Weibin Luo, Chenyang Zhu

**Affiliations:** 1 School of Internet of Things Engineering, Wuxi Taihu University, Wuxi, 214064 China; 2 School of Computer Engineering, Jimei University, Xiamen, 361021 China; 3 School of Computer Science and Artificial Intelligence, Changzhou University, Changzhou, 213000 China; 4 Department of Electronics and Computer Science, University of Southampton, Southampton, SO17 1BJ UK

**Keywords:** Engineering, Mathematics and computing

## Abstract

Fault diagnosis techniques are essential for preventing equipment failures, reducing maintenance costs, and enhancing operational efficiency by promptly identifying anomalies. The widespread deployment of industrial sensors has significantly increased the availability of machinery data, facilitating extensive research in data-driven fault diagnosis. However, real-world datasets frequently exhibit label scarcity and severe class imbalance, where fault instances are substantially fewer than normal samples. To address these challenges, this study proposes a robust unsupervised domain adaptation framework that synthesizes fault signals by interpolating real healthy samples with domain-specific knowledge. Although this synthetic augmentation effectively expands training data, the resulting distribution often deviates from actual fault scenarios, limiting model generalizability. To alleviate this domain discrepancy, our framework incorporates Conditional Domain-Adversarial Networks (CDAN) for domain-invariant feature extraction, complemented by structured pseudo-labeling to assign reliable predictions to unlabeled target samples. Subsequently, a Locality Preserving Projection (LPP) module constructs a shared latent space to achieve both domain alignment and enhanced class discrimination. Experimental evaluations conducted on a synthetic dataset derived from the CWRU bearing benchmark demonstrate that the proposed method achieves accuracies of 91.10% under imbalanced conditions and 84.65% in balanced scenarios, surpassing current state-of-the-art methods by 12.87% and 3.57%, respectively. Ablation studies further underscore the significant contribution of structured pseudo-labeling to the overall performance, confirming the proposed approach’s efficacy and robustness in real-world unsupervised industrial fault diagnosis tasks.

## Introduction

Bearings constitute critical components in numerous industrial rotating machines, including wind turbines, aerospace engines, and high-speed rail systems. Failures in bearings frequently result in extensive equipment damage, operational downtime, and considerable economic losses^1^. Studies have indicated that bearing faults account for up to 50% of failures in large-scale industrial equipment, with this proportion increasing to approximately 90% in smaller mechanical devices^2^. Therefore, accurate and timely bearing fault diagnosis is essential for ensuring operational safety and maintaining production efficiency^3,4^.

Traditional fault diagnosis methods typically depend on mathematical modeling and physical principles. Although effective within their intended contexts, these approaches require significant domain expertise and often exhibit limited applicability beyond specific machinery types or operating conditions. With the rapid development of Industrial Internet of Things (IIoT) and advanced sensing technologies, extensive machinery monitoring data has become increasingly accessible^5^. This proliferation of data has led to the rise of data-driven fault diagnosis approaches, particularly those leveraging deep learning^6–8^.

Deep learning models excel in automatic extraction and classification of abstract representations directly from raw sensor signals. Various deep neural network architectures, including convolutional neural networks (CNNs) and recurrent neural networks (RNNs), have demonstrated superior performance in bearing fault diagnosis tasks^9–11^. For example, Zhang et al^12^. decomposed vibration signals into intrinsic mode functions and utilized a one-dimensional CNN to facilitate end-to-end learning and classification. Lu et al^13^. employed Graham field techniques to convert one-dimensional vibration data into two-dimensional feature representations, thereby enhancing the feature extraction capability of ResNet50 for bearing fault diagnosis. Similarly, Han et al^14^. combined CNN-extracted features with a random forest regression model to improve fault classification accuracy. However, these supervised methods typically rely on extensive labeled fault data, which remains scarce in real-world industrial scenarios due to the infrequency of actual fault occurrences.

In practice, data collected from different operating conditions or environments often exhibit considerable distribution discrepancies, commonly referred to as domain shifts. Such discrepancies significantly degrade the performance of conventional supervised learning methods, as these approaches generally assume identical training and test distributions. To overcome this limitation, unsupervised domain adaptation (UDA) techniques have been increasingly adopted^15^. UDA aims to align the feature distributions of labeled source and unlabeled target domains, enabling models trained on the source domain to generalize effectively to the target domain without requiring additional labeled target samples^16^.

Despite recent advancements, several critical challenges remain unresolved in current UDA-based fault diagnosis approaches. A prominent issue is severe class imbalance in the target domain, where normal (healthy) samples significantly outnumber faulty ones. Additionally, the absence of ground-truth labels often leads to unreliable pseudo-labeling, causing error propagation and compromised classification performance. Previous UDA approaches, such as Domain-Adversarial Neural Networks (DANN)^17^, primarily focus on global domain alignment while neglecting the importance of class-level feature alignment.

To address these challenges, this paper proposes a novel unsupervised fault diagnosis framework named Conditional-Adversarial Alignment with Structured Pseudo-Labeling and Signal Synthesis for Adaptation (CASSA), which integrates Conditional Domain Adversarial Networks (CDAN), Locality Preserving Projection (LPP), and structured pseudo-labeling. Specifically, the proposed method synthesizes fault data by interpolating real healthy signals with expert domain knowledge, effectively alleviating data scarcity and class imbalance. To mitigate distributional differences between synthetic and actual fault samples, CDAN is employed for extracting domain-invariant features, while structured pseudo-labeling progressively assigns high-confidence labels to unlabeled target samples. Subsequently, LPP is adopted to construct a discriminative and domain-aligned latent subspace, further enhanced by introducing Mean Squared Error (MSE) and Minimum Class Confusion (MCC) losses to reduce classification ambiguity^18,19^.

Comprehensive experiments conducted on a synthetic dataset derived from the widely-used Case Western Reserve University (CWRU) bearing dataset demonstrate significant improvements over existing unsupervised fault diagnosis methods, achieving absolute accuracy gains of 12.87% under imbalanced conditions and 3.57% under balanced conditions. Furthermore, ablation studies and sensitivity analyses confirm the critical contributions of structured pseudo-labeling and feature alignment components, highlighting the effectiveness and robustness of the proposed method in realistic industrial fault diagnosis scenarios.

The main contributions of this study are summarized as follows:

1. We propose an unsupervised bearing fault diagnosis framework integrating CDAN and LPP, augmented by structured pseudo-labeling and combined loss functions (MSE and MCC). The framework effectively addresses severe class imbalance and enhances discriminative feature alignment across different domains.
2. To overcome the lack of labeled samples in the target domain, we introduce an iterative structured pseudo-labeling strategy, integrated with MSE and MCC losses, facilitating robust feature learning and progressively refining pseudo-label quality throughout training.
3. Extensive experimental evaluations and ablation studies conducted on the CWRU bearing benchmark dataset demonstrate consistent and substantial improvements in fault diagnosis accuracy over state-of-the-art methods under both balanced and imbalanced conditions. Further analyses confirm the robustness and practical utility of each component within CASSA.

The remainder of this paper is structured as follows: Section “Related work” reviews relevant literature on fault diagnosis and domain adaptation. Section “Proposed framework” details the proposed methodology, followed by experimental results and discussions in Sect. "Experimental results and analysis". Finally, concluding remarks and future work directions are provided in Sect. “Conclusion”.

## Related work

### Fault diagnosis under limited and imbalanced data conditions

Industrial fault diagnosis under scenarios characterized by limited and highly imbalanced data has attracted significant attention due to practical challenges in acquiring sufficient fault samples. Existing strategies to mitigate these issues can be broadly classified into data augmentation, advanced feature representation learning, and classifier optimization.

Data augmentation techniques employing generative models, such as generative adversarial networks (GANs) and variational autoencoders (VAEs), have demonstrated considerable potential. Dixit et al^20^. introduced an enhanced conditional VAE equipped with centroid-based regularization to generate synthetic samples that closely mimic the original fault distribution, thereby enriching minority-class representations. Alternatively, fault signal synthesis through physical modeling and domain expertise represents another viable augmentation strategy. For instance, Wang et al^21^. developed an impulse-based physical model simulating bearing surface defects, subsequently incorporating realistic vibration noise to generate authentic fault samples. Such physics-informed approaches enable construction of representative datasets without extensive reliance on costly experimental fault simulations. In a related line of work, Du et al^22^. proposed a dynamic model-assisted transfer-coupled dictionary learning strategy that integrates physical dynamic modeling with data-driven dictionary learning, allowing the synthesized features to retain domain-invariant dynamics across different operating conditions. This model-informed synthesis mechanism demonstrates that augmenting limited data with physically guided latent structures can substantially improve cross-domain diagnostic consistency.

Beyond data augmentation, recent studies have focused on representation learning under imbalanced and multi-domain conditions. Jian et al^23^. proposed a prototype-guided supervised contrastive learning framework with dynamic temperature modulation, effectively enhancing discriminative feature representation and reducing bias toward majority classes. Similarly, Jian et al^24^. presented a two-stage semi-supervised domain generalization method that integrates pseudo-label refinement with feature alignment to address label scarcity and class imbalance under unknown operating conditions. To further mitigate long-tailed data effects, Jian et al^25^. introduced a long-tailed multi-domain generalization strategy that improves diagnostic robustness under varying working conditions. Earlier work by Jian and Ao^26^employed a semi-supervised ensemble learning framework that adaptively reweights minority-class samples to enhance classification stability. In parallel, Du et al^27^. proposed a dynamic model-driven dictionary learning-inspired domain adaptation approach that adaptively bridges the discrepancy between source and target domains by coupling physical priors with learned representations, effectively reducing bias under limited and imbalanced fault data. These approaches collectively demonstrate that incorporating physical dynamics into representation learning can complement data-driven contrastive and ensemble methods, leading to more interpretable and transferable diagnostic models.

Despite these advances, synthetic and learned feature distributions often exhibit discrepancies compared to genuine fault conditions, which can degrade diagnostic generalization. Consequently, complementary strategies such as domain adaptation and domain generalization have been extensively explored to mitigate distributional shifts in practical industrial environments.

### Adversarial learning-based unsupervised domain adaptation

Adversarial learning frameworks have gained prominence in UDA for fault diagnosis, primarily due to their effectiveness in extracting domain-invariant features. By aligning the feature distributions between labeled source and unlabeled target domains, these methods enhance model generalizability across varied operating conditions^28,29^.

Domain-Adversarial Neural Networks (DANN)^17^ constitute a seminal approach within this paradigm, employing a gradient reversal layer to achieve domain confusion, thereby fostering domain-invariant feature representations. Extending this idea, Conditional Domain-Adversarial Networks (CDAN) integrate class-conditional information, promoting a finer-grained alignment of conditional distributions across domains^30^.

Recent advancements include the Generalized CDAN (GCDAN) framework proposed by Wu et al^31^., which enhances the original CDAN by introducing progressive adaptation mechanisms and auxiliary loss functions, enabling effective handling of multiple fault types and varying operational conditions. Nonetheless, despite their successes, adversarial UDA methods often encounter difficulties under pronounced class imbalance and when pseudo-label accuracy is compromised.

### Pseudo-labeling for domain adaptation

Pseudo-labeling serves as an effective semi-supervised strategy in UDA, leveraging unlabeled target domain data by assigning provisional labels based on model predictions. These pseudo-labels are iteratively refined to progressively improve prediction reliability and classification performance^32^.

However, conventional pseudo-labeling approaches frequently suffer from noise propagation, particularly when model predictions are uncertain or unreliable. To mitigate this, confidence-based selection methods have been proposed. For example, Tanwani et al^33^. introduced a Dirichlet-based confidence evaluation mechanism to retain only the most reliable pseudo-labels for subsequent model training, thus reducing error accumulation. Additionally, Liang et al^34^. enhanced pseudo-label quality by projecting features into a shared latent space and employing maximum likelihood estimation, significantly improving classification robustness in the presence of domain shifts.

In fault diagnosis tasks, pseudo-labeling plays a critical role in bridging distributional gaps between synthetic fault signals and real-world data. Building upon these advancements, our proposed method integrates structured prediction and K-means clustering into a refined pseudo-labeling mechanism. This structured approach assigns high-confidence labels selectively, facilitating enhanced feature alignment and significantly improving model performance in scenarios characterized by severe class imbalance and limited labeled data availability.

## Proposed framework

### Problem formulation

In industrial fault diagnosis, collecting sufficient labeled fault data is often impractical due to the rarity of fault occurrences and the high cost of annotation. To mitigate this issue, synthetic fault data are generated to approximate realistic fault conditions. We denote the labeled synthetic dataset as the source domain $D_S = \{ (u_s^1, v_s^1), \dots , (u_s^m, v_s^m) \}$, where each sample $u_s^i$ has an associated label $v_s^i \in V$, representing one of four bearing states: healthy, inner ring fault (IF), rolling element fault (REF), and outer ring fault (OF). Real-world operational data, obtained without labels, form the target domain $D_T = \{ u_t^1, \dots , u_t^n \}$. We assume that the source and target domains share the same label space but exhibit significant distributional differences caused by operating conditions, noise, and sensor variations. Moreover, the class distribution in the target domain is typically imbalanced, with some fault types underrepresented. The objective of unsupervised fault diagnosis is to construct a model trained only on labeled synthetic source data that can generalize to the unlabeled target data. This requires aligning feature representations across domains while maintaining discriminative power for fault categories. The main challenges include: (1) addressing the distribution shift between synthetic and real data, (2) handling class imbalance in the target domain, and (3) reducing feature divergence caused by variations in working conditions and measurement environments. Our method is specifically designed to tackle these issues.

### Framework overview

**Fig. 1:**
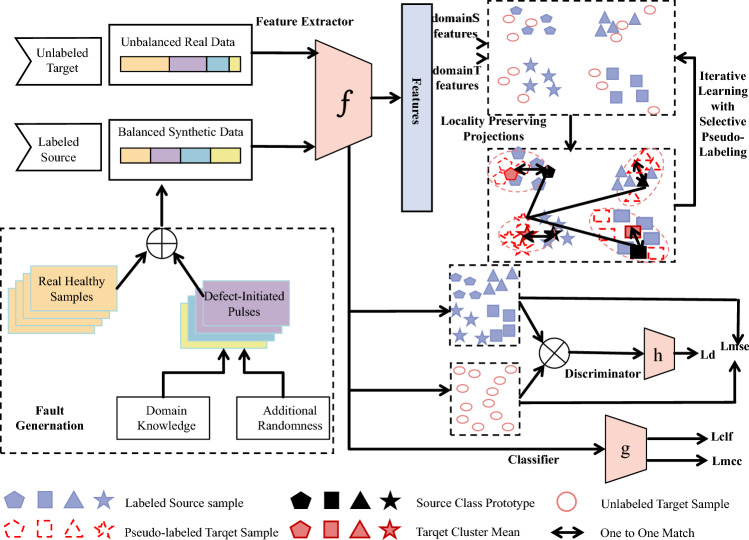
The architecture of the CASSA framework. The core component is the CDAN module. The dashed box in the lower left illustrates the synthetic fault signal generation process, while the dashed boxes in the upper right depict the structured pseudo-labeling and locality preserving feature projection modules.

As shown in Figure 1, the proposed CASSA framework is designed as a progressive two-stage process that integrates conditional domain adaptation and manifold-based structured pseudo-labeling to achieve robust fault diagnosis under limited and imbalanced data conditions. In the first stage, a Conditional Domain-Adversarial Network (CDAN) is employed to learn domain-invariant and discriminative features from labeled synthetic source data and unlabeled real target data. Through adversarial training among a shared feature extractor, a classifier, and a domain discriminator, the model aligns conditional feature distributions between the two domains. To further enhance feature stability and class separability, mean squared error and minimum class confusion losses are incorporated, which regularize feature consistency and reduce overlap between classes. The outcome of this stage is a set of aligned representations that capture both fault-related and domain-independent information.

In the second stage, the normalized features obtained from CDAN are projected into a shared latent subspace using LPP. This projection maintains local geometric relationships while minimizing residual domain discrepancies. Within this subspace, structured pseudo-labeling is performed to assign category labels to target samples. The pseudo-labels are determined by jointly considering the proximity of target samples to source class prototypes and the structural information obtained through clustering in the latent space. High-confidence target samples are selected first to update both the projection and the pseudo-labels, and the process proceeds progressively. As the iterations continue, the subspace representation becomes increasingly discriminative, and the pseudo-labels gain reliability. This progressive refinement ensures that the latent structure and category boundaries are jointly optimized, leading to improved diagnostic generalization across domains.

By combining adversarial alignment in the feature space with progressive pseudo-labeling and projection in the latent subspace, CASSA effectively reduces distribution shift, mitigates class imbalance, and achieves accurate fault classification under real-world operating conditions. Table 1 summarizes the mathematical notations used throughout this paper for clarity.

**Table 1:** List of mathematical notations and corresponding explanations.

**Notation**	**Explanation**
$u_s$, $u_t$	Input samples from the source and target domains, respectively.
$v_s$, $v_t$	True and pseudo labels for source and target samples.
$D_S$, $D_T$	Source and target domain data distributions.
$f_\theta$, $g_\varphi$, $h_\delta$	Feature extractor, classifier, and domain discriminator.
$\mathscr {H}(\cdot )$	Conditioning function for joint feature–label representation.
$\otimes$	Outer product operator used for multilinear mapping.
$\mathscr {M}$	Confusion matrix computed from classifier outputs.
*C*, *V*, |*V*|	Number of classes, class label set, and its cardinality.
$\tilde{U}_s$, $\tilde{U}_t$	Domain-invariant feature representations of source and target samples.
*t*	Gaussian kernel bandwidth in the similarity computation.
$\mathscr {N}_k(\tilde{u}_i)$	Set of *k*-nearest neighbors of sample $\tilde{u}_i$.
$S_{ij}$	Supervision weight matrix encoding label consistency.
*W*, *B*, *L*	Affinity, degree, and Laplacian matrices.
$M \in \mathbb {R}^{d_1\times d}$	LPP projection matrix from original to latent space.
$\alpha$	Regularization coefficient preventing trivial solutions.
$\lambda$	Eigenvalue in the generalized eigenvalue problem.
$Z_s$, $Z_t$	Projected features in the latent subspace.
$\bar{Z}$	Mean of all projected features for mean-centering.
$d(\cdot ,\cdot )$	Euclidean distance function.
$\bar{z}_s^v$, $\bar{z}_t^v$	Source and target class prototypes in latent space.
$m_1(v|\tilde{u}_t)$, $m_2(v|\tilde{u}_t)$	Prototype-based and cluster-based conditional probabilities.
$m(v|\tilde{u}_t)$	Combined conditional probability for pseudo-labeling.
*H*	Binary matching matrix for prototype–cluster assignment.
$n_c$	Initial number of target samples per class for pseudo-label refinement.
*T*	Total number of iterative refinement steps.
$S_l$, $Q_l$	Selected subset and corresponding affinity matrix at iteration *l*.

### Conditional domain-adversarial network

The CDAN serves as the principal domain adaptation mechanism within our framework, consisting of a shared feature extractor $f_\theta$, a classifier $g_\varphi$, and a domain discriminator $h_\delta$. The feature extractor comprises three convolutional layers followed by a fully connected layer. To strengthen feature alignment and classification performance, we augment the standard CDAN objective with Mean Squared Error (MSE) and Minimum Class Confusion (MCC) losses.

The overall optimization objective is formulated as follows:

1 $$\begin{aligned} \min _{\varphi ,\theta }\; \mathscr {L}_{cls}^{s}(u_s,v_s) + \lambda _{adv}\mathscr {L}_{adv}^{s,t}(u_s,u_t) + \lambda _{mse}\mathscr {L}_{mse}^{s,t}(u_s,u_t) + \lambda _{mcc}\mathscr {L}_{mcc}^{t}(u_t), \end{aligned}$$

Here, $u_s$ and $u_t$ denote input samples from the source and target domains, while $v_s$ and $v_t$ represent their corresponding labels (true and pseudo, respectively). The source and target data distributions are denoted by $D_S$ and $D_T$. $\lambda _{adv}$, $\lambda _{mse}$, and $\lambda _{mcc}$ are hyperparameters controlling the respective contributions of each loss term.

The source classification loss ensures accurate fault prediction in the source domain:

2 $$\begin{aligned} \mathscr {L}_{cls}^{s}(u_s,v_s) = -\mathbb {E}_{(u_s^i,v_s^i)\sim D_S}\left[ v_s^i \log g_{\varphi }(f_{\theta }(u_s^i))\right] . \end{aligned}$$

The adversarial loss aligns the conditional distributions of source and target domains:

3 $$\begin{aligned} \begin{aligned} \mathscr {L}_{adv}^{s,t}(u_s,u_t) =&-\mathbb {E}_{u_s^i\sim D_S}\left[ \log h_{\delta }\left( \mathscr {H}(f_{\theta }(u_s^i)\otimes g_{\varphi }(f_{\theta }(u_s^i)))\right) \right] \\&-\mathbb {E}_{u_t^j\sim D_T}\left[ \log \left( 1 - h_{\delta }\left( \mathscr {H}(f_{\theta }(u_t^j)\otimes g_{\varphi }(f_{\theta }(u_t^j)))\right) \right) \right] , \end{aligned} \end{aligned}$$

Here the operator $\otimes$ denotes the outer product between feature and class prediction vectors, and $\mathscr {H}(\cdot )$ represents the conditioning transformation applied to the joint feature–label representation to enhance alignment.

The MSE loss promotes consistency between extracted features and classifier predictions, thus enforcing feature-level regularization across both domains:

4 $$\begin{aligned} \mathscr {L}_{mse}^{s,t}(u_s,u_t) = \mathbb {E}_{u_s^i\sim D_S}\left[ \Vert f_{\theta }(u_s^i)-g_{\varphi }(f_{\theta }(u_s^i))\Vert _2^2\right] + \mathbb {E}_{u_t^j\sim D_T}\left[ \Vert f_{\theta }(u_t^j)-g_{\varphi }(f_{\theta }(u_t^j))\Vert _2^2\right] . \end{aligned}$$

The MCC loss explicitly reduces class confusion among unlabeled target samples, enhancing class separability:

5 $$\begin{aligned} \mathscr {L}_{mcc}^{t}(u_t) = \frac{1}{C}\left( \sum _{i,j=1}^{C}\mathscr {M}_{ij} - \text {trace}(\mathscr {M})\right) , \end{aligned}$$

Here $\mathscr {M}\in \mathbb {R}^{C\times C}$ denotes the confusion matrix of predicted class probabilities for the target domain, and *C* is the total number of fault categories.

After training, the extracted domain-invariant representations from the fully connected layer are denoted as $\tilde{U}_S=\{\tilde{u}_s^1,\dots ,\tilde{u}_s^m\}$ for the source domain and $\tilde{U}_T=\{\tilde{u}_t^1,\dots ,\tilde{u}_t^n\}$ for the target domain. These representations serve as input to the structured pseudo-labeling and LPP modules, further refining diagnostic accuracy.

In summary, the proposed approach strategically integrates adversarial domain adaptation, structured pseudo-labeling, and advanced loss functions to enhance unsupervised fault diagnosis. The combined use of MSE and MCC losses addresses the critical challenges of feature consistency and class confusion, respectively, significantly improving fault classification performance under practical industrial scenarios.

### Structured prediction in latent subspace

Directly training the Conditional Domain-Adversarial Network (CDAN) with synthesized fault datasets frequently leads to discrepancies in class distributions between source and target domains. The underlying assumption of CDAN is that source and target domains share considerable similarity, enabling effective domain alignment through adversarial training. However, when domain divergence is substantial, conventional adversarial alignment alone often becomes inadequate, thus limiting the model’s generalization capabilities. To address this issue, we propose a structured prediction-based pseudo-labeling strategy guided by LPP^35^.

First, the features extracted from source and target domains by the CDAN model undergo L2 normalization, defined as $\tilde{u}\leftarrow \tilde{u}/\Vert \tilde{u}\Vert _2$, to eliminate discrepancies arising from differing feature magnitudes. This normalization ensures that all feature vectors reside on a unified hypersphere, thereby facilitating better alignment. The normalized features from both domains are aggregated into a unified feature matrix $\tilde{U} = [\tilde{U}_s, \tilde{U}_t]$.

Subsequently, we construct a similarity graph to preserve locality structures within the latent feature space. Each data sample is represented as a node, and edges between nodes indicate similarity. The associated affinity matrix *W* is computed via a Gaussian kernel:

6 $$\begin{aligned} W_{ij} = {\left\{ \begin{array}{ll} \exp \left( -\frac{\Vert \tilde{u}_i - \tilde{u}_j \Vert _2^2}{t} \right) & \text {if } \tilde{u}_j \in \mathscr {N}_k(\tilde{u}_i), \\ 0 & \text {otherwise,} \end{array}\right. } \end{aligned}$$

where $\mathscr {N}_k(\tilde{u}_i)$ denotes the set of *k*-nearest neighbors of $\tilde{u}_i$, and the scalar *t* controls kernel bandwidth.

To incorporate class-specific supervision into the similarity structure, we define a supervision weight matrix *S* by leveraging label information:

7 $$\begin{aligned} S_{ij} = {\left\{ \begin{array}{ll} 1 & \text {if } v_i = v_j,\\ 0 & \text {otherwise,} \end{array}\right. } \end{aligned}$$

where $v_i$ and $v_j$ are the labels (or pseudo-labels) of samples *i* and *j*. The supervised similarity matrix is then updated through element-wise multiplication: $W \leftarrow W \odot S$.

Based on the supervised similarity matrix *W*, we define the Laplacian matrix $L = B - W$, where *B* is a diagonal degree matrix with elements $B_{ii} = \sum _j W_{ij}$. The objective of LPP-based dimensionality reduction is thus formulated as:

8 $$\begin{aligned} \min _{M}\frac{\textrm{tr}(M^\top (\tilde{U}L\tilde{U}^\top + \alpha I)M)}{\textrm{tr}(M^\top \tilde{U}B\tilde{U}^\top M)}, \end{aligned}$$

where $M\in \mathbb {R}^{d_1\times d}$ is the projection matrix mapping features from the original space (dimension $d_1$) to a lower-dimensional latent subspace (dimension *d*). The regularization term $\alpha I$ stabilizes the optimization by preventing trivial solutions. Equation (8) can be efficiently solved via generalized eigenvalue decomposition:

9 $$\begin{aligned} (\tilde{U}L\tilde{U}^\top + \alpha I)M = \lambda \tilde{U}B\tilde{U}^\top M, \end{aligned}$$

where the optimal projection matrix *M* consists of eigenvectors corresponding to the smallest eigenvalues.

Once the projection matrix *M* is obtained, features from both source and target domains are projected into the latent subspace *Z* as follows:

10 $$\begin{aligned} Z_s = \tilde{U}_s^\top M,\quad Z_t = \tilde{U}_t^\top M. \end{aligned}$$

To further enhance class separability, mean-centering is applied to the projected features: $Z \leftarrow Z - \bar{Z}$, where $\bar{Z}$ denotes the mean of all projected features. Subsequently, we again apply L2 normalization to the latent features, ensuring robust separability in the projected space.

To generate reliable pseudo-labels, we define class prototypes $\bar{z}_s^v$ as the mean vector of projected source features associated with label *v*:

11 $$\begin{aligned} \bar{z}_s^v = \frac{\sum _{i=1}^{m} z_s^i\,\mathbb {I}(v = v_s^i)}{\sum _{i=1}^{m}\mathbb {I}(v = v_s^i)}, \end{aligned}$$

where $\mathbb {I}(\cdot )$ is the indicator function. We then calculate conditional class probabilities for each target sample based on distance to class prototypes:

12 $$\begin{aligned} m_1(v \mid \tilde{u}_t) = \frac{\exp \left( -d(z_t,\bar{z}_s^v)\right) }{\sum _{v'\in V}\exp \left( -d(z_t,\bar{z}_s^{v'})\right) }, \end{aligned}$$

where $d(\cdot ,\cdot )$ denotes the Euclidean distance.

While nearest-class prototype pseudo-labeling is straightforward, it neglects the intrinsic structure of target domain features. To overcome this limitation, we perform K-means clustering on projected target domain features, initializing clusters with the source class prototypes $\bar{z}_s^v$. Each target cluster centroid $\bar{z}_t^v$ is matched to the closest source class prototype via a one-to-one assignment, formalized as:

13 $$\begin{aligned} \min _{H}\sum _{i=1}^{|V|}\sum _{j=1}^{|V|}H_{ij}\, d(\bar{z}_t^i,\bar{z}_s^j),\quad \text {s.t.}\quad \sum _jH_{ij}=1,\sum _iH_{ij}=1, \end{aligned}$$

where $H\in \{0,1\}^{|V|\times |V|}$ is the matching matrix.

Subsequently, a second set of conditional probabilities for the target samples is computed based on distances to target cluster centroids:

14 $$\begin{aligned} m_2(v \mid \tilde{u}_t) = \frac{\exp \left( -d(z_t,\bar{z}_t^v)\right) }{\sum _{v'\in V}\exp \left( -d(z_t,\bar{z}_t^{v'})\right) }. \end{aligned}$$

To leverage complementary strengths of both prototype-based and clustering-based approaches, we combine the two conditional probabilities as:

15 $$\begin{aligned} m(v \mid \tilde{u}_t) = \max \left\{ m_1(v \mid \tilde{u}_t),\,m_2(v \mid \tilde{u}_t)\right\} . \end{aligned}$$

The final pseudo-label assigned to a target sample is determined by:

16 $$\begin{aligned} \hat{v}_t = \arg \max _{v \in V} m(v \mid \tilde{u}_t). \end{aligned}$$

To further refine pseudo-label quality, we adopt an iterative learning strategy. In each iteration, a subset of high-confidence target samples with assigned pseudo-labels is selected per class, progressively enlarging the set across iterations. Formally, for each category $c\in V$, we initially select $n_c$ target samples and progressively increase the subset size to $k n_c/T$, where *T* denotes the total number of iterations. Through repeated refinement of pseudo-labels and the latent projection matrix *M*, our structured prediction approach effectively alleviates domain divergence and enhances diagnostic accuracy in unsupervised industrial fault diagnosis.

The proposed iterative learning framework based on selective pseudo-labeling is outlined in Algorithm 1. It systematically integrates structured pseudo-label generation, LPP, and progressive refinement steps to address domain divergence and class imbalance inherent in UDA for fault diagnosis.

Specifically, the iterative process commences by normalizing the extracted source features $\tilde{U}_s = \{ \tilde{u}_s^1, \dots , \tilde{u}_s^m \}$ and target features $\tilde{U}_t = \{ \tilde{u}_t^1, \dots , \tilde{u}_t^n \}$ to eliminate scale discrepancies, facilitating improved domain alignment. Initially, we learn the latent projection matrix $M_0$ through LPP, utilizing labeled source domain features and their corresponding labels $V_S = \{ v_s^1, \dots , v_s^m \}$. Based on this initial projection, preliminary pseudo-labels for target features are assigned according to the structured prediction strategy described in Equation (16), considering both prototype-based similarity and clustering-based structural information.

In each iteration *l* ($l = 1, \dots , T$), the algorithm progressively refines the pseudo-labels and latent space projections through a structured subset selection approach. Specifically, the algorithm selects a subset $S_l \subseteq \tilde{U}_t$ containing $\frac{l n^c}{T}$ samples per category $c \in V$ based on the confidence scores derived from the current pseudo-labels $\hat{V}_l$. Subsequently, an affinity matrix $Q_l$ is computed to reflect the local similarity structures and updated pseudo-label information as shown in Equation (6). Leveraging this affinity matrix, the projection matrix $M_l$ is refined through generalized eigenvalue decomposition according to Equation (9), further aligning features across domains and enhancing intra-class compactness.

Upon updating the latent projection matrix, the algorithm recalculates pseudo-labels for all target samples using the updated representation, ensuring iterative enhancement of pseudo-label quality and alignment effectiveness. The iterative optimization continues until the algorithm reaches the pre-specified maximum number of iterations *T*, resulting in a robust and discriminative latent representation that significantly improves fault classification performance in the target domain.

**Algorithm 1:**
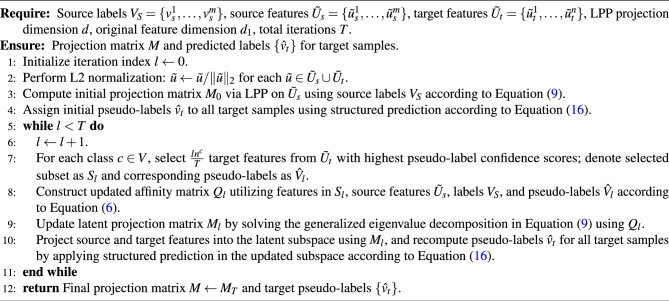
Iterative Structured Pseudo-labeling Framework

## Experimental evaluation

To evaluate the efficacy of the CASSA framework, we conducted extensive experiments on a synthetic dataset derived from the well-known CWRU bearing dataset. The synthesized dataset integrates domain-specific knowledge and realistic fault-type characteristics to ensure its representativeness for real-world industrial conditions. The experimental analyses comprehensively encompass baseline comparisons, ablation studies, sensitivity analyses of key hyperparameters, and evaluations under both balanced and imbalanced data distributions. Experimental results consistently demonstrate that the proposed approach achieves superior generalization and robustness when transferring diagnostic knowledge from synthetic training samples to practical fault diagnosis scenarios.

### Synthetic dataset generation from the CWRU benchmark

To rigorously assess the domain adaptation capability of our framework, we constructed a synthetic dataset based on the standard CWRU bearing dataset, widely employed as a benchmark in fault diagnosis research. The synthesized dataset consists of four distinct fault classes: healthy, inner ring fault (IF), rolling element fault (REF), and outer ring fault (OF). Raw vibration signals were recorded from an accelerometer positioned at the drive-end bearing at a sampling frequency of 12 kHz. During preprocessing, each vibration signal was segmented into multiple segments of length 4096 points, from which 100 segments per class were randomly selected. Subsequently, we applied Butterworth band-pass filtering, signal rectification, and envelope extraction via low-pass filtering. The envelope signals were then transformed into frequency domain spectra through Fourier transform and normalized, yielding a final feature representation of 1200 dimensions for each sample.

To ensure balanced fault representations in the source domain, synthetic fault samples were generated following the procedure outlined by Wang et al^21^.. Specifically, fault signals were synthesized by systematically embedding realistic fault signatures into baseline healthy signals through a controlled interpolation process. This synthesis approach preserves intrinsic structural information and variability, thereby facilitating effective domain adaptation to real target conditions.

The synthetic fault signal generation process utilizes real vibration data acquired from healthy bearings, formulated as follows:

17 $$\begin{aligned} \eta (t)=\sum _{i=-\infty }^{\infty } B_i S(t-iT)+\rho \, m(t), \end{aligned}$$

where $\eta (t)$ denotes the synthetic vibration signal capturing periodic impacts caused by bearing defects occurring at period *T*. The term *S*(*t*) represents an impulse waveform shaped by a Hann window with a 5% duty cycle relative to the fundamental period *T*. Background noise *m*(*t*) is sampled from actual healthy bearing recordings and scaled by a random factor $\rho$, uniformly drawn from [0.25, 2], ensuring realistic variations. The amplitude modulation $B_i$ of each impulse is defined as:

18 $$\begin{aligned} B_i=\alpha \sum _{p=0}^{P}\nu _p\cos {\left( \frac{2\pi \, i\, p\, T}{Q}\right) }, \end{aligned}$$

where $\alpha$ is a random variable with mean 1 and standard deviation 0.1, introducing amplitude variability. The parameter $\nu _p$ controls the number and magnitude of sidebands observed in the envelope spectrum, providing realistic fault signature characteristics. Notably, the values of $\nu _p$ were selected empirically and were not specifically tuned to individual operating conditions.

### Experimental setup and parameter configuration

All experiments were implemented using TensorFlow and MATLAB on a single-processor Intel(R) Core(TM) i5-7300HQ CPU (2.5 GHz). This configuration highlights that the proposed CASSA framework can be executed efficiently without reliance on high-performance GPUs. The training procedure consisted of two consecutive stages corresponding to the CDAN-based domain alignment and the structured pseudo-labeling refinement. In the first stage, both DANN and CDAN models were trained end-to-end for 100 epochs with a batch size of 128 and an initial learning rate of 0.001 using the Adam optimizer. The adversarial, mean-squared-error, and minimum-class-confusion loss terms in Equation (1) were weighted equally, that is, $\lambda _{adv}=\lambda _{mse}=\lambda _{mcc}=1$. The CDAN feature extractor contained three convolutional layers followed by a fully connected layer of 256 units with ReLU activations and batch normalization. The classifier comprised one hidden layer of 128 units and a softmax output, and the domain discriminator was a two-layer multilayer perceptron with 256 and 128 units, respectively.

After CDAN training, domain-invariant source and target features were normalized and projected into a latent subspace through LPP with dimension $d=128$. The LPP regularization coefficient was set to $\alpha =10^{-3}$, the neighborhood size was $k=5$, and the Gaussian-kernel bandwidth *t* was fixed at 1. Structured pseudo-labeling was then carried out iteratively for $T=15$ refinement steps. Each iteration selected high-confidence target samples to update both the projection and pseudo-labels, progressively improving alignment quality.

The imbalanced target dataset was generated from the CWRU benchmark by varying per-class sample counts: 1200 healthy samples, 120 outer-ring-fault (OF) samples, 60 inner-ring-fault (IF) samples, and 12 rolling-element-fault (REF) samples. The balanced dataset contained 1200 samples per class. For both scenarios, all source samples were used for adaptation and all target samples were used for testing. This configuration ensures fair and reproducible evaluation across balanced and imbalanced label distributions while maintaining computational feasibility on CPU-only hardware.

## Experimental results and analysis

### Comparative performance analysis against state-of-the-art methods

Table 2 reports the comparison of UDA methods on the CWRU bearing dataset under imbalanced and balanced target-label conditions. CASSA attains the highest accuracy in both settings, reaching 91.10% under imbalance and 84.65% under balance. In addition to accuracy, the proposed framework also achieves the best Macro Recall, Macro F1, and G-mean in both regimes, demonstrating its ability to maintain balanced recognition performance across all classes. Under the imbalanced setting, CASSA records 91.06% Macro Recall, 90.78% Macro F1, and 89.81% G-mean, surpassing the next best baseline, DASSL^36^, by 7.12%, 7.12%, and 7.43%, respectively. Under the balanced setting, CASSA achieves 84.67% Macro Recall, 84.46% Macro F1, and 83.24% G-mean, showing consistent improvement over CMD+SP (84.30%, 84.00%, and 82.90%) by 0.37%, 0.46%, and 0.34%. These results indicate that the proposed approach is not only accurate in the easier balanced regime but also maintains strong class-level robustness under skewed label distributions, which is common in real-world fault diagnosis.

**Table 2:** Comparative evaluation of different UDA methods on the CWRU dataset under balanced and imbalanced conditions. Besides accuracy, we report recall, F1, and G-mean to provide a more comprehensive evaluation.

Method	Imbalanced				Balanced			
	Accuracy	Macro Recall	Macro F1	G-Mean	Accuracy	Macro Recall	Macro F1	G-Mean
Source Only	61.40%	60.90%	59.10%	53.40%	61.40%	61.20%	60.70%	59.80%
DANN^17^	67.85%	67.10%	65.20%	62.30%	79.00%	78.80%	78.30%	77.00%
CDAN^30^	73.88%	73.50%	71.90%	69.00%	76.52%	76.30%	75.90%	74.80%
CORAL^37^	72.46%	72.20%	70.60%	68.00%	80.88%	80.70%	80.30%	79.20%
CORAL + SP	76.15%	75.94%	74.29%	70.87%	84.40%	84.27%	84.03%	82.77%
CMD^38^	66.31%	65.70%	63.70%	60.90%	79.92%	79.80%	79.40%	78.20%
CMD + SP	73.25%	72.90%	71.40%	68.70%	84.46%	84.30%	84.00%	82.90%
ACDANN^21^	78.23%	77.90%	76.80%	74.90%	81.08%	80.90%	80.60%	79.50%
DASSL^36^	83.94%	83.94%	83.66%	82.38%	80.52%	80.40%	80.10%	78.90%
CASSA	**91.10%**	**91.06%**	**90.78%**	**89.81%**	**84.65%**	**84.67%**	**84.46%**	**83.24%**

Relative to classical adversarial and discrepancy-based methods, the improvements are consistent across all metrics. For example, the proposed method exceeds DANN^17^ by 23.25% in accuracy, 23.96% in Macro Recall, and 27.51% in G-mean under the imbalanced case, and by 5.65%, 5.87%, and 6.24% under the balanced case. It also outperforms CORAL^37^ by 18.64% in accuracy, 18.86% in Macro Recall, 20.18% in Macro F1, and 21.81% in G-mean under imbalance, while maintaining margins of 3.77%, 3.97%, 4.16%, and 4.04% under balance. The results for Macro Recall and G-mean confirm that CASSA effectively mitigates the bias toward majority classes and balances sensitivity and specificity. Although variants such as CORAL+SP and CMD+SP alleviate class imbalance by reweighting samples, their gains remain moderate compared with CASSA, which jointly addresses domain and class-level misalignment.

The consistent superiority of the CASSA across all four metrics indicates that its design directly addresses two central challenges in UDA for fault diagnosis: domain shift and label skew. Under imbalanced target distributions, the high Macro Recall and G-mean demonstrate that the framework successfully reduces the impact of label shift and class-conditional mismatch, both of which commonly bias decision boundaries toward dominant classes. Under balanced conditions, the method achieves stable performance across accuracy, Macro Recall, and F1, confirming its ability to extract domain-invariant and class-discriminative features without overfitting to source priors. The observed results align with strategies that integrate class-conditioned alignment, target-prior correction, and confidence-regularized pseudo-labeling, leading to consistently higher performance under both regimes.

The contrast between balanced and imbalanced conditions highlights the relative weaknesses of existing UDA methods in maintaining per-class fairness. Most baselines experience a noticeable decline in Macro Recall and G-mean when target labels become imbalanced, showing that global alignment strategies like DANN^17^, CDAN^30^, CORAL^37^, and CMD^38^ remain vulnerable to skewed label distributions. The inclusion of rebalancing components in variants slightly improves their metrics but still falls short of CASSA. In contrast, CASSA exhibits nearly identical trends between accuracy and Macro F1, and its high G-mean further supports that both majority and minority classes are well aligned. The framework thus provides a comprehensive balance between accuracy and class-level consistency, a key requirement for reliable UDA in industrial fault diagnosis.

### Ablation analysis of framework components

**Table 3:** Ablation study on the contribution of each component in the proposed framework. The results show that CDAN provides effective feature extraction, while the combination of LPP with structured pseudo-labeling (SP) and K-nearest neighbor (KNN) refinement further improves subspace alignment and overall adaptation performance.

Framework components				CWRU (Imbalanced)	CWRU (Balanced)
Baseline	CDAN	SP	KNN	Accuracy	Accuracy
$\surd$	$\times$	$\times$	$\times$	61.40%	61.40%
$\surd$	$\surd$	$\times$	$\times$	73.88%	76.52%
$\surd$	$\surd$	$\surd$	$\times$	65.30%	78.60%
$\surd$	$\surd$	$\surd$	$\surd$	**91.10%**	**84.65%**

The ablation results in Table 3 clarify the individual and combined effects of the major modules in the proposed framework. The framework is composed of two key stages: (1) feature extraction through CDAN and (2) subspace alignment using LPP, which is further enhanced by structured pseudo-labeling (SP) and K-nearest neighbor (KNN) refinement.

Starting from the baseline model trained solely on source data, the performance is limited to 61.40% accuracy under both balanced and imbalanced conditions. Introducing CDAN substantially improves the results to 73.88% and 76.52%, confirming its ability to extract domain-invariant features through conditional adversarial training. This step effectively aligns marginal distributions between the synthetic source and real target domains, forming a strong foundation for subsequent subspace adaptation.

In the second stage, LPP is applied to align the extracted CDAN features in a unified subspace. When SP is introduced to guide this process, the performance increases to 78.60% under balanced conditions but decreases to 65.30% under imbalance. This reflects the sensitivity of SP to skewed class distributions: although SP encourages structural consistency by preserving relationships between target samples, it can amplify class bias when pseudo-labels are unreliable or unevenly distributed.

The integration of KNN refinement within the LPP stage addresses this limitation by imposing local consistency among neighboring samples in the subspace. KNN correction mitigates label noise from SP and enhances the stability of alignment by enforcing smooth decision boundaries around locally similar target samples. When both SP and KNN are jointly applied, accuracy rises dramatically to 91.10% and 84.65% under imbalanced and balanced conditions, respectively. This result verifies that SP provides global structural regularization, while KNN ensures local coherence, producing a balanced and noise-resilient alignment across domains.

Overall, the ablation analysis demonstrates that CDAN effectively captures transferable features, and the LPP-based alignment module, which is strengthened by SP and KNN, further refines the adaptation process. Their combined effect not only bridges the global domain gap but also improves local discriminability, resulting in robust cross-domain fault diagnosis performance under both balanced and imbalanced scenarios.

### Impact of class imbalance on diagnostic performance

**Fig. 2:**
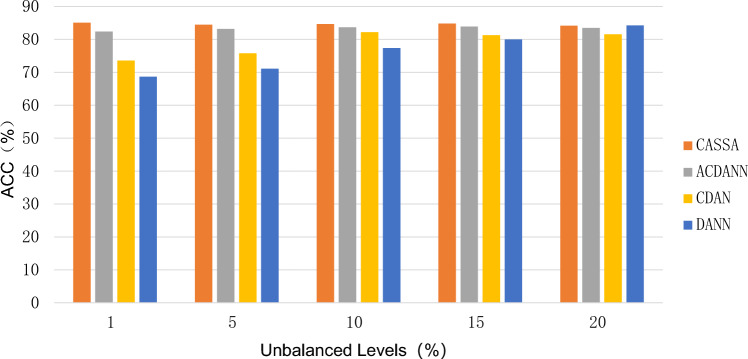
Impact of varying class imbalance levels on diagnostic accuracy, specifically adjusting the proportion of Rolling Element Fault (REF) samples in the target domain. Performance comparison among DANN, CDAN, and the proposed structured prediction-based framework highlights robust performance of our method under severe imbalance conditions.

Figure 2 presents the diagnostic accuracy of different domain adaptation methods under varying levels of class imbalance in the target domain, where imbalance severity is controlled by adjusting the proportion of Rolling Element Fault samples. The results demonstrate clear differences in the robustness of DANN, CDAN, ACDANN, and CASSA when subjected to increasingly skewed class distributions. As the proportion of REF samples decreases to extreme imbalance levels (1% and 5%), DANN and CDAN exhibit marked performance degradation, achieving only 68.7% and 73.6% accuracy at the 1% level, respectively. Although their performance improves as the imbalance becomes less severe, the fluctuation across levels reflects their sensitivity to skewed label distributions. By contrast, CASSA consistently delivers high accuracy, maintaining above 84% even under the most extreme imbalance, thereby highlighting the stability of structured prediction in handling distributional asymmetries. ACDANN performs moderately well, with less severe degradation than CDAN and DANN, yet its overall performance remains below that of CASSA across all imbalance levels.

The degradation observed in DANN and CDAN under severe imbalance can be attributed to their reliance on global adversarial alignment. Both frameworks primarily align marginal feature distributions without explicitly addressing class-conditional discrepancies. In scenarios where minority fault classes such as REF are underrepresented, adversarial training tends to prioritize majority classes, leading to biased decision boundaries and compromised generalization. This vulnerability is further amplified by the reliance on pseudo-labels generated from imbalanced target data, which are more likely to misclassify minority samples, thereby propagating errors during adaptation.

In contrast, the proposed CASSA framework demonstrates remarkable robustness against imbalance. By incorporating structured prediction into the adaptation process, CASSA enforces consistency not only at the global distribution level but also across class-conditional structures. This mechanism effectively mitigates the dominance of majority classes and ensures that minority fault types, including REF, are adequately represented in the learned decision boundaries. Consequently, the framework preserves diagnostic accuracy across all imbalance levels, outperforming competing methods under both extreme and moderate imbalance conditions.

The strength of structured prediction lies in its ability to stabilize pseudo-labeling through the integration of relational constraints between classes. By capturing inter-class dependencies, CASSA reduces the risk of noisy supervision and prevents the collapse of minority class representations. Specifically, for rare fault types such as REF, structured prediction enhances the reliability of pseudo-labels by leveraging contextual relationships among fault categories, thereby improving class-conditional alignment and maintaining robustness against skewed distributions. This design addresses a key limitation of conventional adversarial methods, which lack explicit mechanisms to safeguard minority classes.

These findings carry broader implications for unsupervised domain adaptation in industrial fault diagnosis. In practical applications, class imbalance is a common and often severe challenge, as some fault types occur rarely but remain critical for reliable machinery monitoring. The superior performance of CASSA under extreme imbalance suggests that adaptation strategies must go beyond marginal alignment and incorporate mechanisms that explicitly account for label skew and noisy supervision. The results further emphasize that methods relying solely on adversarial training are insufficient in highly imbalanced industrial scenarios, reinforcing the importance of hybrid frameworks that balance global distribution matching with structured class-level refinement.

### Sensitivity analysis of key framework parameters

**Fig. 3:**
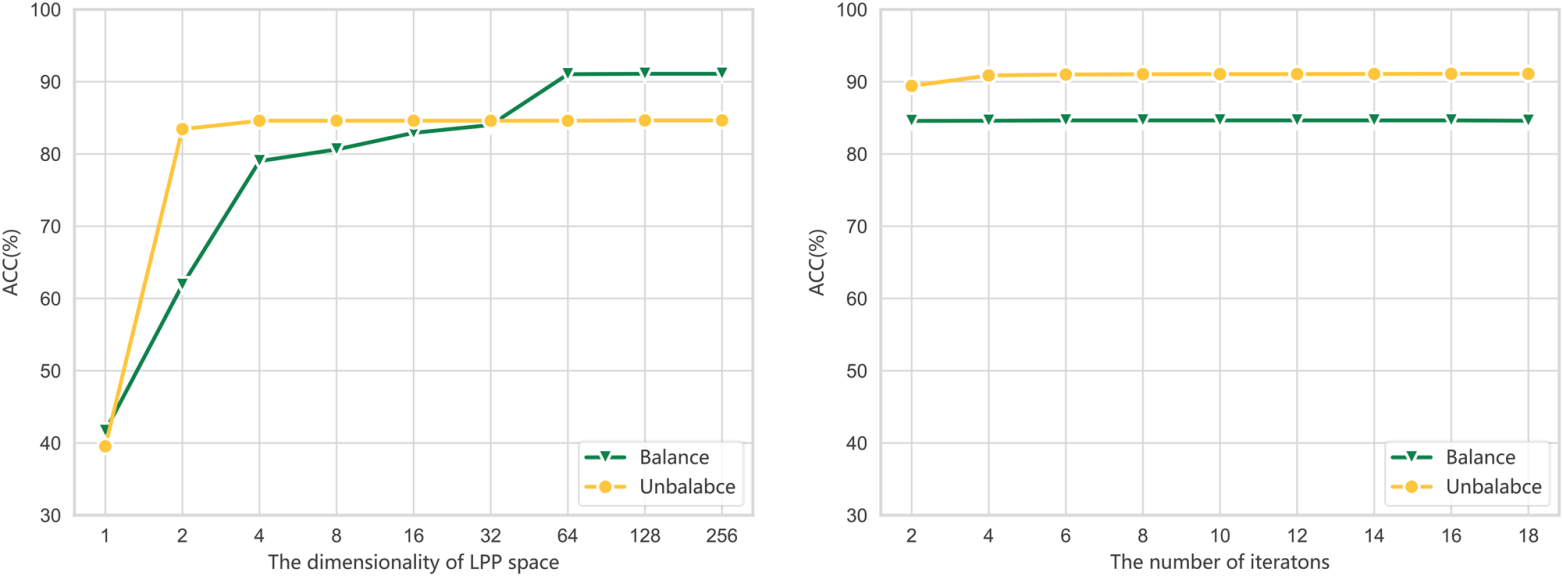
Sensitivity analysis of (**a**) projection dimensionality (*d*) and (**b**) iterative refinement rounds (*T*) in balanced and imbalanced scenarios.

Figure 3 and the accompanying quantitative results provide a sensitivity analysis of two central parameters in the proposed framework: the number of iterative refinement steps (*T*) and the latent subspace dimensionality (*d*). The results highlight that both parameters influence diagnostic accuracy, yet the framework remains stable across a broad range of values, underscoring its robustness and practical deployability in industrial fault diagnosis.

For the refinement steps (*T*), the results indicate a clear pattern of initial performance gains followed by convergence to a stable plateau. In the imbalanced setting, accuracy improves substantially from 87.31% at $T=1$ to 90.96% at $T=5$, reaching 91.10% by $T=15$, after which no further gains are observed. A similar trend emerges in the balanced case, where performance increases marginally from 84.56% at $T=1$ to 84.65% at $T=6$, saturating thereafter. These results demonstrate that iterative refinement enhances performance by progressively reducing domain misalignment and stabilizing pseudo-labels, but excessive iterations yield diminishing returns due to convergence saturation. Beyond a certain threshold, further refinement introduces the risk of over-smoothing, where excessive updates reduce the discriminative sharpness of decision boundaries, thus providing negligible or no performance improvements.

The analysis of latent subspace dimensionality (*d*) reveals a complementary perspective. Extremely small dimensions, such as $d=1$, result in poor performance with accuracies of 41.67% (imbalanced) and 39.54% (balanced), indicating insufficient representation capacity for capturing the variability of fault characteristics. Increasing *d* improves performance substantially, with accuracy rising to 79.02% (imbalanced) and 83.44% (balanced) at $d=4$. The best results are observed when *d* is moderately large: at $d=64$, the framework achieves 91.04% under imbalance, while balanced performance stabilizes around 84.60%. Further increases in dimensionality to $d=128$ or $d=256$ yield negligible gains, suggesting that excessively high-dimensional subspaces may induce overfitting or introduce redundancy, thereby limiting generalization capability. This trade-off illustrates the existence of an optimal range for *d* that balances discriminative expressiveness with the ability to generalize effectively across domains.

### Threat of validity

Despite the clear advantages of CASSA, certain limitations remain. While the framework maintains stable performance across imbalance levels, its absolute accuracy does not continue to increase with higher proportions of REF samples, indicating a potential ceiling effect in leveraging additional balanced data. Moreover, the reliance on structured prediction introduces additional computational overhead, and its sensitivity to the quality of initial pseudo-labels may still impact performance in highly noisy domains. Future extensions may explore imbalance-aware reweighting strategies, adaptive confidence thresholds, or hybrid refinement mechanisms to further improve resilience under extreme skew. Such refinements could enhance both efficiency and robustness, enabling broader deployment of structured adaptation in complex industrial fault diagnosis settings.

## Conclusion

This paper presents an innovative unsupervised fault diagnosis framework that integrates CDAN with a structured pseudo-labeling strategy to simultaneously address domain shift and enhance fault classification performance. Experimental validation on the CWRU dataset confirms the effectiveness of our approach, achieving an accuracy of 91.10% over leading state-of-the-art methods. An additional advantage of our framework lies in its robustness to unknown levels of class imbalance commonly encountered in practical industrial applications. Even under balanced fault conditions, our method achieves an accuracy of 84.65%, outperforming existing models. Moreover, the conducted ablation experiments highlight the critical roles played by both the CDAN module and structured prediction in improving model generalization and reliability. Overall, the proposed architecture not only demonstrates strong diagnostic capabilities in synthetic-to-real adaptation scenarios but also lays the groundwork for future research into more adaptive and scalable unsupervised learning techniques in industrial fault detection.

## Data Availability

The datasets analysed during the current study are available in the Bearing Data Center, https://engineering.case.edu/bearingdatacenter/.

## Code availability

The code will be made available after the manuscript is accepted.
